# The Frequency and Patterns of Post-COVID-19 Vaccination Syndrome Reveal Initially Mild and Potentially Immunocytopenic Signs in Primarily Young Saudi Women

**DOI:** 10.3390/vaccines10071015

**Published:** 2022-06-24

**Authors:** Kamaleldin B. Said, Amal Al-Otaibi, Luluh Aljaloud, Basmah Al-Anazi, Ahmed Alsolami, Fayez Saud Alreshidi, on behalf of the Ha’il COM Research Unit Group

**Affiliations:** 1 Department of Pathology and Microbiology, College of Medicine, University of Ha’il, Ha’il 55476, Saudi Arabia; s201806142@uoh.edu.sa (A.A.-O.); s201803639@uoh.edu.sa (L.A.); s201800476@uoh.edu.sa (B.A.-A.); 2 Genomics, Bioinformatics and Systems Biology, Carleton University, 1125 Colonel-By Drive, Ottawa, ON K1S 5B6, Canada; 3 ASC Molecular Bacteriology, McGill University, 21111 Lakeshore Rd., Montreal, QC H9X 3L9, Canada; 4 Department of Internal Medicine, College of Medicine, University of Ha’il, Ha’il 55476, Saudi Arabia; a.alsolami@uoh.edu.sa; 5 Deparmtent of Family, Community Medicine, College of Medicine, University of Ha’il, Ha’il 55476, Saudi Arabia; fs.alreshidi@uoh.edu.sa

**Keywords:** COVID-19 reactions, ITP syndromes 2, COVID-vaccine women susceptibility

## Abstract

Vaccination is the most promising approach for ending or containing the SARS-CoV-2 pandemic. However, serious post-COVID-19 vaccine reactions, including immunocytopenia (ITP) syndrome, have been increasingly reported. Several factors cause increased risks including multiple doses, age-dependent heterogeneity in immune-responses, platelet cross-reactions with microbial components, and Long-COVID syndrome. Thus, in the absence of widely available specific therapeutics, vigilance is important while more studies are needed. Using a structured questionnaire sent to different regions in Saudi Arabia, we conducted a comprehensive investigation on the frequency, rates, disease patterns, and patient demographics of post-COVID-19 vaccine side effects on febrile patients after administration three major vaccines. Results indicated that the majority of respondents administered Pfizer BioNtech vaccine (81%, *n* = 809); followed by AstraZeneca (16%, *n* = 155); and Moderna (3%, *n* = 34). Overall 998 participants, 74% (*n* = 737) showed no serious symptoms; however, 26.2% (*n* = 261) revealed typical syndromes. In a focused group of 722 participants, the following rates were identified: shortness of breath (20%), bruises or bleeding (18%), inattention (18%), GIT symptoms (17.6%), skin irritation (8.6%), and anosmia and ageusia (8%) were the most prominent among those who showed typical symptoms. The onset time was mostly between 1–3 days in 49% (*n* = 128), followed by 4–7 days in 21.8% (*n* = 57), 8–14 days in 16.5% (*n* = 43), and more than a month in 12.6% (*n* = 33). The onsets occurred mostly after the first, second, or both doses, 9%, 10%, and 7% of participants, respectively. The frequency of symptoms was significantly higher after Moderna^®^ vaccine (*p*-value = 0.00006) and it was significantly lower in participants who received Pfizer (*p*-value = 0.00231). We did not find significant difference in symptoms related to differences in regions. Similarly, the region, age, sex, education, and nationality had no influence on the dose and onset timings. The findings of this study have significant clinical implications in disease management strategies, preventive measures, and vaccine development. Future vertical studies would reveal more insights into the mechanisms of post-COVID-19 vaccine syndrome.

## 1. Introduction

The global community is struggling to recover in the aftermath of the most devastating coronavirus pandemic in recent history caused by the Severe Acute Respiratory Syndrome Coronavirus (SARS-CoV-2). In a short time, the virus spread worldwide, dramatically changing human behaviors. Fortunately, in a massive effort to control the virus, there have been a total of 11,655,356,423 vaccine doses administered as of 9th May 2022 globally (WHO coronavirus COVID-19 dashboard, available online at https://covid19.who.int/ accessed 13 May 2022). This makes it the most rapid and extensive vaccination campaign in history that employed state-of-the-art mRNA vaccine products in human history, the Pfizer Biontech, Moderna, and AstraZeneca, and others. However, as with all vaccines, this did not go without side effects; despite their high efficacy and safety, cases of potential side effects including increased risk of mucocutaneous or major bleeding were reported [1]. One of the most prominent side effects recorded was a condition that first appeared in different reports as a prothrombotic syndrome. It has been described differently as vaccine-induced immune thrombotic thrombocytopenia (VITT), thrombosis with thrombocytopenia syndrome (TTS), and vaccine-induced prothrombotic immune thrombocytopenia [2]. Generally, it is an autoimmune disease characterized by isolated platelet count <100 × 10^9^/L, episodes of possible hemorrhage caused by antiplatelet antibodies, and petechiae or purpuric rashes [3]. However, in the absence of exposure it was named VITT. According to American Society of Hematology, VITT is defined as a clinical syndrome characterized by positive antibodies against platelet factor 4 (PF4) identified by enzyme-linked immunosorbent assay (ELISA) assay. The casual relationship of ITP to post-COVID-19 vaccine injection has not been well defined [4]. Therefore, it is not yet fully clear whether all are vaccine-induced secondary ITP or incidental primary ITP that occurred soon after vaccination [5]. As with other countries, reports on incidence of this side effect are limited in the Middle Eastern countries.

Despite the reported high safety and efficacy of COVID-19 vaccines, some potential side effects remain, including autoimmune responses. However, detailed review on all aspects of this condition is limited. For instance, a systematic review on 45 new onsets of ITP post-COVID-19 vaccination showed, after excluding other concomitant factors that can trigger thrombocytopenia in COVID-19, that 75% cases were moderate-to-severe and that the majority of ITP cases (71%) appeared in senior patients (>50 years), while only three were pediatric cases (7%) [6]. In the aforementioned study, 20% of cases of ITP symptoms rose three weeks after onset of COVID-19 symptoms, and no bleedings were reported in 31% cases at diagnosis [6]. Appearance of ITP post-COVID-19 vaccine has been reported in different countries worldwide including USA [7], Mexico [8], Italy [9], and UK [10]. The ITP is known to occur mainly in adults, particularly young women. This suggests that sex hormones, as in other immune disorders such as systemic lupus and multiple sclerosis, may play a role in the susceptibility to ITP. The condition first appeared in different reports as a prothrombotic syndrome in a small number of individuals after AstraZeneca vaccine administration (University of Oxford, and Serum Institute of India), an adenoviral vector-based vaccine [11]. Subsequently, similar findings were observed in a small number of individuals who received the Ad26.COV2.S vaccine (Janssen; Johnson & Johnson), also based on an adenoviral vector [12]. Similarly, sporadic cases were reported after Pfizer and Moderna vaccines; however, the rate of ITP was 0.80 per million doses for both vaccines [13]. Lee et al. [14] reported a series of cases of very low platelet counts occurring within two weeks of Pfizer and Moderna vaccinations upon review of published data from the USA CDC, the Food and Drug Administration (FDA), agencies of the U.S. Department of Health and Human Services (HHS), Vaccine Adverse Events Reporting System (VAERS) [15,16]. These included 20 case reports on ITP after vaccination, 17 reports without pre-existing ITP, and 14 with reported bleeding symptoms prior to hospitalization. The aforementioned authors also reported that 19 of 20 hospitalized patients aged 22–73 years (11 females and 8 males) showed petechiae, bruising, or mucosal bleeding in 1–23 days post Pfizer (9 patients) and Moderna (11 patients) vaccination, along with platelet counts mostly ≥10 × 10^9^/L (range 1–36 × 10^9^/L; median 2 × 10^9^/L). The vaccine-induced ITP could possibly have similar pathogenicity to COVID-19 vaccine-induced DIC, which highlights the importance of further investigation in agreement with others [17]. Recently, a new phenomenon characterized by ITP was reported in multiple patients after vaccination with the ChAdOx1 nCoV-19. The adverse effects in this case remained exceptionally low following the vaccine of more than 400 million people. This new syndrome was quite similar to heparin-induced ITP [18]. Detailed studies characterizing the rates, frequencies, and patterns of occurrence, as well as pathogencity of these cases is limited.

COVID-19 vaccines can trigger a series of unique ITP-related syndromes including skin reactions, shortness of breath, inattention, gastrointestinal (GIT) symptoms, anosmia, and ageusia. Molecular mimicry between SARS-CoV-2 spike-protein and human microbial components may potentially elicit adverse skin and other pathological reactions post vaccinations. For instance, Gambichler et al. reported on most frequent early reactions due to vaccination are known to occur at injection-sites; for instance, Type I, Type IV hypersensitivity reactions including delayed large local skin lesions popularly dubbed as (“COVID arm”) can occur [19]. In addition, reactions in dermal filler, previous radiation sites, or even old BCG scars, and more frequently morbilliform and erythema multiforme-like rashes and different forms of autoimmune-mediated skin conditions post-COVID-19 vaccination are likely to occur. Functional angiopathies, pityriasis rosea-like rashes and reactivation of herpes zoster have been also reported after COVID-19 vaccination [20]. Similarly, Catala et al. studied 405 reactions from 16 February to 15 May 2021 following vaccination with the BNT162b2 (Pfizer-BioNTech; 40.2%), mRNA-1273 (Moderna; 36.3%), and AZD1222 (AstraZeneca; 23.5%) vaccines. Patient means were 50.7 years and 80.2% of them were female. Cutaneous reactions were COVID arm (32.1%), urticaria (14.6%), morbilliform (8.9%), papulovesicular (6.4%), and pityriasis rosea-like (4.9%) and purpuric (4%) reactions. Varicella zoster and herpes simplex virus reactivations were reported as 13.8% of reactions. The most frequent reported reactions in each vaccine group were COVID arm (mRNA-1273, Moderna, 61.9%), varicella zoster virus reactivation (BNT162b2, Pfizer-BioNTech, 17.2%), and urticaria (AZD1222, AstraZeneca, 21.1%). The COVID arm was almost exclusive to females (95.4%). Most reactions to the mRNA-1273 (Moderna) vaccine were described in females (90.5%). Eighty reactions (21%) were classified as severe and 81% needed specific treatment [21]. Thus, COVID-19 patients with ITP are at an increased risk of mucocutaneous or major bleeding [1]. In addition to skin reactions, dyspnea and wheezing were identified as the earliest signs of thrombocytopenia syndrome [22]. In fact, it has been well established that severe fever, although not easily characterized, has been directly linked to early signs of ITP syndrome in several cases [23,24,25]. In addition, it has also been known that immune ITP is more than a bleeding disorder; cognitive symptoms are commonly reported [26]. Furthermore, the cognitive impairments leading to brain fog, a newly emerging post-COVID-19 manifestation, has been hypothesized post-mRNA vaccinations [27]. An association between COVID-19 vaccine (ChAdOx1, AstraZeneca^®^, Cambridge, UK) and cerebral vein thrombosis was reported. New reports emerged on the association of the aforementioned effects with mortality and long-term morbidity. The VTE was reported in unusual locations following the ChAdOx1 vaccine, resulting in its suspension in several countries. Consequently, 169 cases of cerebral vein thrombosis (CVT) and 53 cases of splanchnic vein thrombosis were reported to the European Medicines Agency (EMA) among 35 million ChAdOx1 vaccine recipients [28,29]. However, a population cohort study in Denmark and Norway reported increased rates of venous CVT among recipients of the ChAdOx1 vaccine with no increase in arterial events [30]. These recent data suggested an excess rate of CVT of 2.5 per 100,000 ChAdOx1 recipients, although laboratory testing has not confirmed that they were due to vaccine VITT [31]. Thus, data on the cognitive effect(s) of COVID-19 are also limited.

Additional hypersensitivity reactions for overexpression of type I interferons, COVID-19-induced coagulopathy, thrombotic microangiopathy, and direct viral damage were suggested as side effects. In addition, delayed reactions at injection sites were also observed for the mRNA-1273 vaccine clinical trial (onset after day 8) in 0.8% participants after the first dose and in 0.2% after the second dose [32]. In a retrospective analysis of the effects of SARS-CoV-2 vaccination on 109 ITP patients identified with preexisting ITP, approximately 20% experienced an ITP exacerbation following the first dose with 14 of 70 patients having an exacerbation after the second dose. Response to treatment and outcomes were also favorable in the patients with preexisting ITP, and no major bleeds were reported after vaccination. Therefore, the authors concluded that ITP might worsen in some patients with preexisting ITP or may occur de novo post-SARS-CoV-2 vaccination.

Although there is an ongoing risk of ITP after administration of many other vaccines including influenza, measles-mumps-rubella (MMR), hepatitis B, human papilloma virus, varicella, and diphtheria-tetanus-pertussis (DPT) vaccines in children and adolescents [33,34,35], the COVID-19 reaction carries an additional layer of risk due to several factors. Unlike other viral vaccines, the COVID-19 received multiple booster doses. In addition, there is risk of age-dependent heterogeneity in SARS-CoV-2-immune responses in senior patients [36]. Furthermore, there is a high possibility that initially mild reactions progress into severe prolonged symptoms known as “long-COVID”, leading to multisystem failure and disability. More important, the disseminated intracellular coagulation (DIC) and the consequence of thrombocytopenia has been reported as a major viral mechanism in COVID-19 in contrast to cytokine storm [37]. Thus, in the absence of a widely available specific therapeutics, caution and more post-COVID-19 vaccination studies have become imperative. The aim of this study was to conduct a comprehensive investigation on the frequency, rates, disease patterns, and patient demographics on post-COVID-19 side effects with emphasis on ITP.

## 2. Materials and Methods

### 2.1. Study Design

The current study was conducted as a retrospective cross-sectional survey using a self-administered structured online questionnaire through the Google platform.

### 2.2. Study Population

All individuals who had taken COVID-19 vaccines and who agreed to participate in the study, aged  ≥  18 years, and living in Saudi Arabia were eligible to participate. We posed no restrictions on the sex, nationality, occupation, or socioeconomic level of the participants. However, the emphasis was placed on those with severe fever and fatigue patients.

### 2.3. Data Collection Tool

A self-administered computer-based multiple choice questionnaire was used. An online link of the web-based survey was developed in Google to obtain data regarding side effects of COVID-19, with emphasis on high-grade fever and signs of ITP from October to December 2021. On the first screen of the questionnaire, a Plain Language Information Statement (PLIS) and consent to participate were enclosed. Any details for contact or personal data that could identify the participant were not used. Contact details of the study investigators were given in the PLIS for transparency. Only the participants with fever and providing consent to participate in the study were permitted to move to the next section containing the screening questionnaire to confirm. Only participants who confirmed the predefined age-limit were moved to the next pages containing the self-administered survey. The questionnaire consisted of 18 questions, including 8 general questions about the respondent, 5 questions related to the vaccine, and 5 more related to general history. All responses were analyzed for significant findings on the prevalence rates in the country. Participants’ answers to questions directly related to side effects were transferred into analysis scores.

### 2.4. The Questionnaire: Development and Validation of the Questionnaire

In the process of developing the questionnaire, a wide comprehensive review of the available literature about the ITP was performed. Subsequently, a thorough review on the available data, relevancy, vaccine types, and profiles of individuals and disease patterns were studied. A focused discussion was conducted by experts and the final version was used. This was validated for content, criterion, and construct components. For further evaluation, a pilot study of 30 participants was performed, where different reliability measures were also tested, including test-retest reliability/repeatability, consistency, and inter-rater reliability.

### 2.5. Statistical Analysis

Data were analyzed using IBM SPSS for Windows version 26 statistical software (Statistical Package for Social Sciences (SPSS) software version 26 (SPSS Inc., Chicago, IL, USA). Categorical data were reported as frequency/percentage and continuous data as mean/standard deviation. The analysis was descriptive and stratified; we presented absolute numbers, proportions, and graphical distributions. We conducted exact statistical tests for proportions and showed *p*-values where appropriate (a *p*-value < 0.05 was considered statistically significant).

## 3. Results

Since response to vaccine is a multifactorial issue affected by numerous factors, we carefully examined several host, vaccine, and ecological factors including nationality, region of residence, educational level, vaccine type, dose, onset time, previous medical history, and age and sex differences. Main leading responses included were fatigue and highly febrile participants with sings of ITP. However, 998 participants, 74% (*n* = 737) had no bruises or bleeding ITP symptoms; these typically appeared in 26.2% of highly febrile participants (*n* = 261) as explained below. We did not find significant differences in the frequency of bruises and bleeding ITP symptoms related to differences in regions of Saudi Arabia. Similarly, no significant differences were found between regions, ages, and sexes, in the onset timings as well as in the dose numbers after which typical syndromes appeared. The findings of this study have significant clinical implications in disease management strategies, preventive measures, and vaccine development.

**Figure 1:**
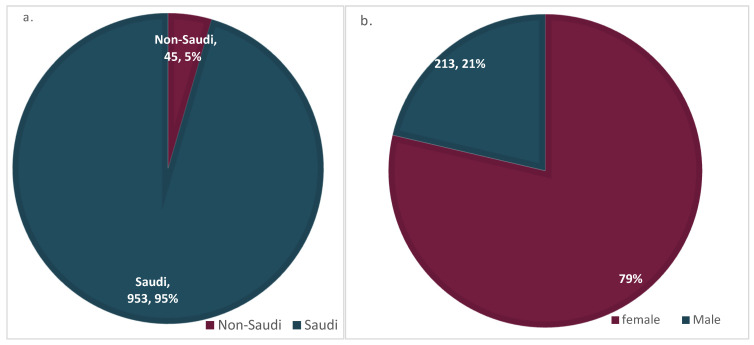

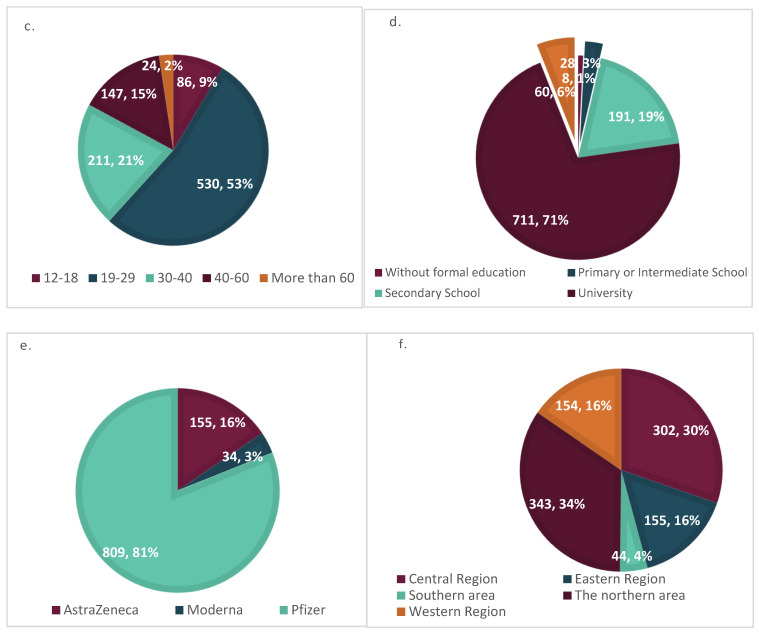
(**a**) Nationality-specific response to post-COVID-19 vaccine issues in Saudi Arabia. (**b**) Sex-specific response to post-COVID-19 vaccine issues in Saudi Arabia. (**c**) Age-specific response to post-COVID-19 vaccine issues in Saudi Arabia. (**d**) Education-specific response to post-COVID-19 vaccine issues in Saudi Arabia. (**e**) Overall vaccine-type administered by respondents in the study. (**f**) Region-specific respondents to post-COVID-19 vaccine issues in Saudi Arabia.

In this study, education, nationality, age, and sex differences in response to different vaccines administered at different regions in Saudi Arabia were as shown below (Supplementary materials). Among the 998 participants, the overwhelming majority administered the Pfizer BioNTech vaccine followed by AstraZeneca and Moderna vaccines (81%, *n* = 809; 16%, *n* = 155; and 3%, *n* = 34, respectively) (Table 1). These participants were primarily young (19–29 years old 53.1%, *n* = 530), educated (71%, *n* = 711), Saudi (95%, n = 953), and female (78.7%, *n* = 785) (Figure 1a–f). The rates of different age groups among participants were as follows: 12–18 years old 8.6% (*n* = 86), 19–29 years old 53.1% (*n* = 530), 30–40 years old 21.1% (*n* = 211), 40–60 years old 14.7% (*n* = 147), and seniors 60 years or more comprised 2.4% (*n* = 24). However, respondents’ estimates based on region showed that most were from the Northern and Central regions (34.4%, *n* = 343; 30.3%, *n* = 302, respectively) followed by similar rates found in Eastern (15.5%, *n* = 155) and Western regions (15.4%, *n* = 154); whereas, the Southern region had the least responders with 4.4% (*n* = 44) (Figure 1f). Furthermore, respondents based on educational levels showed that among the 998 participants, 71.2% (*n* = 711) had a university degree, while 19.1% (*n* = 191) were secondary school certificate holders, 6.0% (*n* = 60) had postgraduate degrees, 2.8% (*n* = 28) had school certificates, and 0.8% (*n* = 8) had no formal education (Figure 1d).

**Figure 2:**
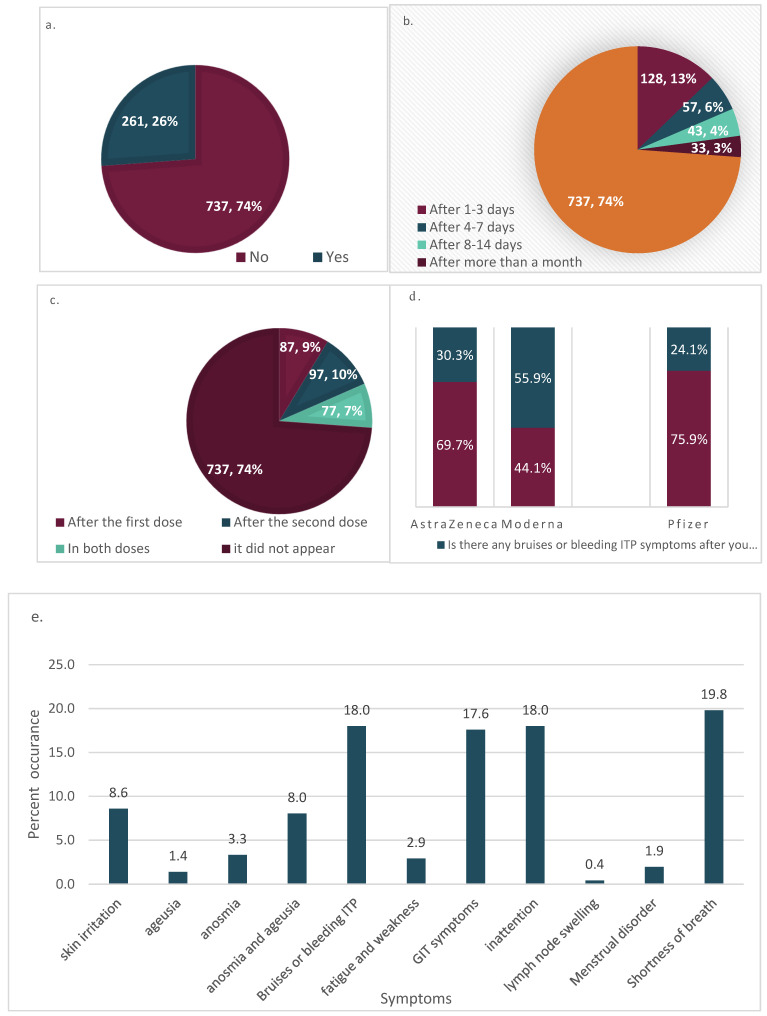
(**a**) Frequency of bruises or bleeding symptoms with high fever post any COVID vaccine administered in Saudi Arabia. (**b**) Overall onset times of ITP symptoms post-COVID vaccine in Saudi Arabia. (**c**) COVID-19 vaccine dose-dependent bruises and bleeding in Saudi Arabia. (**d**) COVID-19 vaccine type-dependent typical ITP symptoms in Saudi Arabia. (**e**) Frequencies of direct ITP and other symptoms related to vaccine interaction.

We monitored the frequency of visible syndromes in response to different vaccine including ITP symptoms, which was highly significant. In 998 participants, 74% (*n* = 737) had no bruises or bleeding ITP symptoms albeit they had other common manifestations. However, typical bruises or bleeding ITP symptoms associated with high fever appeared in 26.2% of participants (*n* = 261) as shown in Figure 2a. For those in whom typical symptoms appeared, the onset of symptom times ranged from 1–3 days in 49% (*n* = 128), 4–7 days in 21.8% (*n* = 57), 8–14 days in 16.5% (*n* = 43), and in 12.6% (*n* = 33) of participants the onset was within more than a month (Figure 2a,b and Table 2). This indicated that the risk of ITP was more within the first three days. Based on the dose-dependent symptoms, the signs appeared in 9%, 10%, and 7% after the first, second, and both doses, respectively (Figure 2c). Finally, the frequency of bruises and ITP symptoms was significantly higher among participants who received Moderna^®^ (*p*-value = 0.00006) and it was significantly lower in participants who received Pfizer (*p*-value = 0.00231) (Figure 2d). However, there was no significant difference in the frequency of bruises and bleeding ITP symptoms between different regions of Saudi Arabia. Similarly, no significant differences were associated with differences in regions, sexes, and age groups in the onset times (in days) as well as in the doses after which and bleeding ITP symptoms occurred.

After removal of common symptoms such as transient low-grade fevers and headache and focusing on questions from respondents about direct ITP-related symptoms associated with sever fever, 722 out of 998 cases were analyzed. In this group, although the overall rates for side effect(s) were acceptably low, the following reactions were reported: shortness of breath (19.88%), bruises or bleeding (18%), inattention (18%), GIT symptoms (17.6%), skin irritation (8.6%), and anosmia and ageusia (8%) were the most prominent among respondents (Figure 2e).

## 4. Discussion

The COVID-19 mass vaccination campaign has been unique in the 21st century in speed, efficacy, and approach. This campaign employed the first in-silico biologics used directly from bench to arm. The efficacy of the mRNA vaccines was among the highest seen in history, albeit their serious side effects made it imperative for safety and efficacy to be revisited. In this comprehensive study, we reported on specific post-vaccine syndromes with emphasis on direct ITP reactions known to be induced by vaccine component(s). To cover the wide breadth of potential factors that are prone to influence post COVID-19 side effects, we included a variety of questions including nationality, region of residence, educational level, vaccine type, onset time, previous history, and age and sex differences. However, in 998 participants, 74% (*n* = 737) had no bruises or bleeding ITP symptoms. Typical bruises or bleeding ITP symptoms appeared in 26.2% of participants (*n* = 261) as shown above. We did not find significant difference in the frequency of symptoms in the different regions of Saudi Arabia. Similarly, no significant differences were found between regions, sexes, and age groups, in onset days as well as in the doses after which bleeding ITP symptoms occurred. The findings of this study have significant clinical implications in disease management preventive measures, and vaccine development strategies.

The finding that respondents were primarily young (19–29 years old 53.1%, *n* = 530), educated (71%, *n* = 711), Saudi (95%, *n* = 953), female (78.7%, *n* = 785) is a significant criterion indicating age- and sex-specific factors in susceptibility to post-COVID vaccine reactions. Our results are consistent with recent studies on similar sample sizes of studies in different continents including Germany, Israel, and Africa [38,39,40]. This implies that being female at a young age is a major global risk factor that is constant across different nations with mosaic as well as homogenous population genetic structures. The common global factor widely known is that the ITP occurs mainly in young adults, particularly women in their third or fourth decade with an overall female-to-male ratio of 3–4 to 1. These figures suggest that sex hormones, as in other immune disorders such as systemic lupus and multiple sclerosis, may play a role in the susceptibility to ITP. More importantly, global genetic variability in susceptibility to post vaccine reactions might have no significant role since we obtained similar results in this genetically homogeneous population in Saudi Arabia. Thus, several gaps exist in the pathogenicity, clinical profiles, and preventive measures of SARS-CoV-2. Since regional variations in respondents did not alter the common patterns of syndromes seen, it is plausible that larger-scale national surveys would potentially reach to the same present conclusions we reported here.

Significant data on post-COVID vaccination syndrome are required. Vigorous profiling of post-vaccine syndrome has become imperative in the absence of a widely available specific therapeutic, the risk of age-dependent heterogeneity in SARS-CoV-2-immune responses [36], and the high possibility that initially mild reactions progress into severe prolonged symptoms known as “long-COVID”, leading to multisystem failure and disability. In this study, the overall frequency of side effects was low albeit some were serious. We found 26.2% (*n* = 261) of respondents showed side effects mostly occurring after the firsts dose in 33.3% (*n* = 87) within the first three days and declined afterwards, potentially indicating an initial immune trigger in the vaccine. It has been shown that several early immune signatures, including plasma RIG-I levels, early interferon signaling, and related cytokines (CXCL10, MCP1, MCP-2, and MCP-3) associated with subsequent disease progression, control of viral shedding, and the SARS-CoV-2-specific T cell and antibody response measured up to several months after activation [41].

It is not clear why the frequency of ITP-related symptoms was significantly higher among participants who received Moderna^®^ (*p*-value = 0.00006) but was significantly lower in those who received Pfizer (*P*-value = 0.00231). This observation occurred even though the overwhelming majority administered the latter vaccine. Rashes and skin reactions to Moderna vaccine related reactions have been reported in seniors. For instance, a case of purpuric rash and thrombocytopenia was reported in a 60-year-old comorbid African American male in the USA after the first dose of the m-RNA-1273 vaccine [42]. Similarly, a 66-years-old obese Guyanese male presented with a bullous rash following receipt of a commercial COVID-19 mRNA vaccine [43]. The US Food and Drug Administration (FDA) has granted emergency use authorization for the Pfizer/BioNTech and Moderna COVID-19 vaccines to protect recipients from a SARS-CoV-2 infection by formation of antibodies and provide immunity against a SARS-CoV-2 infection. However, while both vaccines can cause various adverse effects, they are comparatively more frequent after Moderna COVID-19 vaccine, which is stored at lower temperatures for ease of transportation [44]. Although reactions to post Moderna vaccination are commonly reported in comorbid seniors, we reported on this reaction in primarily young (19–29 years old 53.1% (*n* = 530)) females (78.7%, *n* = 785) of Saudi nationality (95%, *n* = 953) without underlying causes.

Menstrual bleeding was one of the least prevalent symptoms we reported in this study with only 1.9% of women experiencing it. However, consistent with earlier findings and contrary to unsupported beliefs, bleeding is not generally proportional to the platelet count. In earlier studies in adults with ITP cases with a platelet count of less than 50 × 10^9^/L, the presenting symptom was hemorrhage in 12% and purpura in 58% [45] while 28% remained asymptomatic and in other cases more than half of patients remained so with a platelet count of 30 to 50 × 10^9^/L [46]. Albeit ITP has been widely known as a bleeding disorder, many findings indicate otherwise. For instance, ITP has been found paradoxically associated with thrombosis; a 4-year follow up in United Kingdom revealed thromboembolism incidence was about 1.3 times higher in patients with ITP than in matched controls [47]. More important is that ITP is potentially associated, in many cases, with common microbial infections necessitating pre-diagnosis. The antigenic cross-reactivity between organisms as Hepatitis C virus, HIV, and *Helicobacter pylori* with platelet glycoproteins can elicit antibody response against platelets leading to thrombocytopenia [48,49]. Finally, we report on significant gastrointestinal symptoms post-COVID-19 vaccination; almost 18% of patients revealed GIT signs mostly during the first week. It was not clear whether those were cytokine release syndrome (CRS) that are known to induce ITP. For these reasons, definitive diagnosis of common underlying causes for ITP is important before attributing effects to vaccines per se. Patients with stomach and colorectal cancers have been reported to have CRS evidenced by raised inflammatory markers, thrombocytopenia, elevated cytokine levels (IFN-γ/IL-2R/IL-18/IL-16/IL-10), and steroid responsiveness [50]. This particularly becomes complicated when patients have on-going infections as Hepatitis C virus, HIV, and *H. pylori* that can induce cross-reacting antibodies against platelets intiating ITP symptoms. Prospects on treating ITP by eradicating *H. pylori* have been reported [51]. A review on pathophysiological interaction between platelets and pathogens, as well as the clinical consequences of platelet dysregulation discussed several points. These included that the occurrence of thrombocytopenia during sepsis or septic shock, considered a well-known risk factor for disease severity. Furthermore, sepsis-induced thrombocytopenia can be associated with several changes, including an altered morphological pattern, receptor expression, and aggregation which can occur even with a normal platelet count and able to modify host response and the severity of the infection [51]. Therefore, a great deal of certainty and definitive diagnosis to exclude cancers, microbial infections, and any potential underlying causes that can aggravate post-COVID-vaccine syndromes has become imperative before attributing any effect to vaccines per se. The limitation of this study is that it used online questionnaire and that a much more widely covered comprehensive study including all cities and regions on a much longer-time scale might give more insights into the issues of post vaccination effect(s).

## 5. Conclusions

For the first time in the Middle East and perhaps globally, and to the best of our knowledge, we report on the COVID-19 vaccine related typical signs on initially highly febrile and potentially immunocytopenic young, educated, primarily Saudi females 1–3 days after administration of mRNA vaccines. The majority of respondents administered Pfizer BioNtech vaccine followed by AstraZeneca and Moderna. Fortunately, most had no immediate serious symptoms. However, the first prevalence of the initially mild syndromes in febrile patients at specific onset times post administration revealed typical patterns of progressions into potentially immunocytopenic signs that warrant subsequent vertical studies. The frequency of symptoms was significantly higher after Moderna^®^ vaccine (*p*-value = 0.00006) and it was significantly lower in participants who received Pfizer (*p*-value = 0.00231). The absence of significant difference in symptoms between substantially different geographic regions and the seemingly independent dose and onset timings of region, age, sex, education, and nationality implied a common response to a universal vaccine component(s). We further recommend that, since several factors aggravate post-vaccine immune reactions including multiple doses, age-dependent heterogeneity in immune-responses, platelet cross-reactivity to pathogen antigens, and long-COVID syndrome, vigorous screening tests for definitive results should become imperative before conclusions on any case. The findings of this study have highly significant clinical implications in disease management strategies, preventive measures, and more importantly in advanced synthetic biologics development. Future vertical studies on reverse-vaccinology, genome-based virus-host interactions against the local population’s genetic structure would potentially reveal more insights into the mechanisms of post-COVID vaccine syndrome. Thus, in the absence of widely available specific therapeutics, vigilance is important while more studies are needed.

## Figures and Tables

**Table 1:** Frequency of different types of vaccines administered by respondents in the study.

	Frequency		Percent	Valid Percent	Cumulative Percent
Valid	AstraZeneca	155	15.5	15.5	15.5
	Moderna	34	3.4	3.4	18.9
	Pfizer	809	81.1	81.1	100.0
	Total	998	100.0	100.0	

**Table 2:** Overall frequency of bruises or bleeding ITP symptoms and onset times after any COVID-19 vaccine in Saudi Arabia.

Onset Times after Symptoms						Overall Frequency of Bruises or Bleeding ITP Symptoms					
		Frequency	Percent	Valid Percent	Cumulative Percent			Frequency	Percent	Valid Percent	Cumulative Percent
Valid	After 1–3 days	128	12.8	12.8	12.8	Valid					
	After 4–7 days	57	5.7	5.7	18.5						
	After 8–14 days	43	4.3	4.3	22.8		No	737	73.8	73.8	73.8
	After > month	33	3.3	3.3	26.2		Yes	261	26.2	26.2	100.0
	it did not appear	737	73.8	73.8	100.0						
	Total	998	100.0	100.0			Total	998	100.0	100.0	

## Data Availability

All data included in the manuscript, Supplementary Materials are attached along with this submission.

## Supplementary Materials

The following supporting information can be downloaded at: https://www.mdpi.com/article/10.3390/vaccines10071015/s1, Supplementary file: Results—CoVID vaccine study12022022.
